# Disparities in Risks of Malaria Associated with Climatic Variability among Women, Children and Elderly in the Chittagong Hill Tracts of Bangladesh

**DOI:** 10.3390/ijerph17249469

**Published:** 2020-12-17

**Authors:** Theophilus I. Emeto, Oyelola A. Adegboye, Reza A. Rumi, Mahboob-Ul I. Khan, Majeed Adegboye, Wasif A. Khan, Mahmudur Rahman, Peter K. Streatfield, Kazi M. Rahman

**Affiliations:** 1 Public Health & Tropical Medicine, College of Public Health, Medical & Veterinary Sciences, James Cook University, Townsville, QLD 4811, Australia; theophilus.emeto@jcu.edu.au; 2 Australian Institute of Tropical Health and Medicine, James Cook University, Townsville, QLD 4811, Australia; 3 International Centre for Diarrhoeal Disease Research, Bangladesh (icddr,b), Dhaka 1212, Bangladesh; rumi@icddrb.org (R.A.R.); khanmahboob@yahoo.com (M.-U.I.K.); wakhan@icddrb.org (W.A.K.); pkstreatfield@icddrb.org (P.K.S.); 4 American University of Nigeria, 640001 Yola, Nigeria; majeed.adegboye@aun.edu.ng; 5 Institute of Epidemiology, Disease Control and Research (IEDCR), Dhaka 1212, Bangladesh; mahmudur57@gmail.com; 6 North Coast Public Health Unit, New South Wales Health, Lismore, NSW 2480, Australia; Kazi.Rahman@health.nsw.gov.au; 7 The University of Sydney, University Centre for Rural Health, Lismore, NSW 2480, Australia

**Keywords:** climatic variability, malaria, vulnerable groups, Chittagong Hill Tracts, Bangladesh

## Abstract

Malaria occurrence in the Chittagong Hill Tracts in Bangladesh varies by season and year, but this pattern is not well characterized. The role of environmental conditions on the occurrence of this vector-borne parasitic disease in the region is not fully understood. We extracted information on malaria patients recorded in the Upazila (sub-district) Health Complex patient registers of Rajasthali in Rangamati district of Bangladesh from February 2000 to November 2009. Weather data for the study area and period were obtained from the Bangladesh Meteorological Department. Non-linear and delayed effects of meteorological drivers, including temperature, relative humidity, and rainfall on the incidence of malaria, were investigated. We observed significant positive association between temperature and rainfall and malaria occurrence, revealing two peaks at 19 °C (logarithms of relative risks (logRR) = 4.3, 95% CI: 1.1–7.5) and 24.5 °C (logRR = 4.7, 95% CI: 1.8–7.6) for temperature and at 86 mm (logRR = 19.5, 95% CI: 11.7–27.3) and 284 mm (logRR = 17.6, 95% CI: 9.9–25.2) for rainfall. In sub-group analysis, women were at a much higher risk of developing malaria at increased temperatures. People over 50 years and children under 15 years were more susceptible to malaria at increased rainfall. The observed associations have policy implications. Further research is needed to expand these findings and direct resources to the vulnerable populations for malaria prevention and control in the Chittagong Hill Tracts of Bangladesh and the region with similar settings.

## 1. Introduction

Malaria, a febrile life-threatening illness, caused by the malaria parasites transmitted primarily by the vector *Anopheles* mosquitoes [1], is endemic in Bangladesh [2]. There is seasonal and inter-annual variation in malaria occurrence affecting part of the country, including the Chittagong Hill Tracts [2,3]. Evidence suggests an increasing incidence of vector-borne diseases, including malaria, in Bangladesh in the last few decades [4]. The annual malaria incidence increased from 1556 cases in 1971 to 15,375 cases in 1981 [5]. Again from 1991 to 2004, the number of reported cases rose from 30,282 to 42,012 cases [5].

The role of the environment in the occurrence of vector-borne diseases is increasingly of interest in recent decades [6,7]. Vector-borne diseases have been differentially associated with climatic conditions globally [8,9,10]. A direct influence of interannual and interdecadal climate variability on the epidemiology of vector-borne diseases has been proposed [8]. The continuing change in climatic conditions and its impact on human civilization, including human health, is now central to the epidemiologic factors linked to malaria [9,10]. Malaria is a key endemic vector-borne disease in South East Asia [4,11]. This region and specifically Bangladesh is considered to be the most vulnerable to climate change, and thus sensitive to vector-borne diseases in the long-term [12,13,14,15]. Knowledge of the modes of transmission of vector-borne diseases such as malaria was said to be critical in implementing effective preventive measures and eradication [9,14]. Malaria distribution and the human population at risk of infection have been suggested to expand proportionally with a change in climatic factors [16]. Research demonstrates an association between malaria incidence and meteorological factors globally [9,17,18,19,20].

Worldwide, there was approximately 0.6 °C increase in the average surface temperature (two-thirds of which occurred post-1975) and a drastic change in the pattern of rainfall and humidity in the last century [21]. Similar changes in the climate have also been experienced in Bangladesh [22]. There is a strong association between climatic conditions and vectors, such as mosquitoes [12,23]. Increased rainfall and humidity increases vector breeding grounds and hence their density [24,25]. Increased temperature increases the biting rate and shortens the external incubation period of mosquitoes [24,26].

Although, the association between meteorological variables and the incidence of malaria have been generally established, the behavioural interactions between these variables and the demographics varies across regions. This variation is perhaps due to climate change, shifts in geographical locations suitable for transmission, or differing seasonal lengths/activities [16,27,28,29]. Additionally, the intensity of malaria transmission vary spatially and temporally depending on environmental fluctuations and specific vector-parasite combinations [30]. Nevertheless, there are still gaps in the general knowledge of meteorological-malaria relationships in Bangladesh. Therefore, we hypothesized that the increasing occurrence of malaria in Bangladesh could be explained, at least partly, by climate change or climatic variability in the country. We aimed to investigate the association between climatic variability and the occurrence of malaria in south-eastern Chittagong Hill Tract district Rangamati in Bangladesh. We used distributed lag non-linear models (DLNMs) [31] to simultaneously explore the non-linear and lag effects of temperature, relative humidity, and rainfall on the incidence of malaria. We further assessed whether the associations varied by age and sex in the subgroup analyses.

## 2. Materials and Methods

### 2.1. Study Site and Study Population

**Figure 1:**
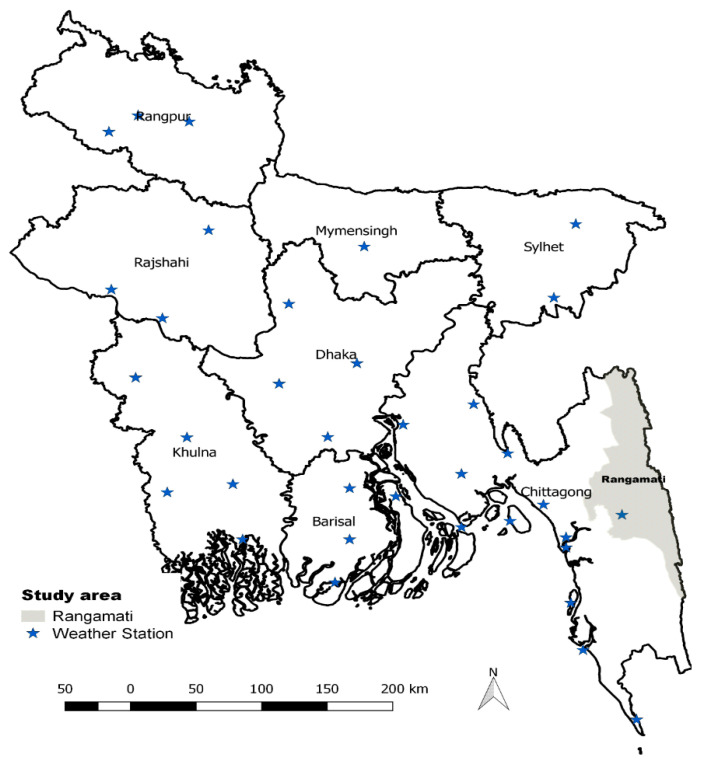
Bangladesh map showing the study area (Rangamati subdistrict shaded in grey) and available weather stations.

The study was conducted in Rangamati district of Chittagong division in Bangladesh (Figure 1). Rangamati has one of the highest malaria burdens in the country [11]. The district has ten sub-districts. The study population comprised the general population of Rajasthali sub-district, having three unions: Bangalhalia, Ghila Chhari, and Gainda.

### 2.2. Ethical Approval

We collected information on diagnosed malaria patients from the health facility record. All the patient information were de-identified before analysis. No individual interviewing was conducted. The Research Review Committee and Ethical Review Committee of International Centre for Diarrhoeal Disease Research, Bangladesh (icddr,b) approved the research design and data collection before conducting the study (protocol number: PR-09013).

### 2.3. Data Sources and Study Variables

The outcome of interest was the occurrence of malaria among the study subdistricts in Rajasthali. A case of malaria was diagnosed by a qualified health care provider and supported by microscopic examination. Study staff extracted information on malaria cases from the patient records in the Rajasthali Upazila (sub-district) Health Complex (UHC). A structured proforma was used for recording the data. All malaria patients who reported to the UHC from February 2000 to November 2009 were included in the analysis.

Meteorological data, recorded in the weather station in Rangamati (Figure 1), were obtained from the Bangladesh Meteorological Department (BMD). Meteorological data on temperature, rainfall, and relative humidity were included in the analyses as exposure variables. The temperature was measured in degree centigrade (°C), rainfall in millimetres (mm), and relative humidity in percentage (%).

### 2.4. Statistical Analysis

Both daily meteorological and malaria data were aggregated to mean weekly data due to sparse daily counts. The associations between weekly time-series counts of confirmed malaria cases and weekly meteorological factors (temperature, relative humidity, and rainfall) were investigated using a generalized linear model (GLM) with a distributed lag and non-linear dependencies [31]. It is very common in time-series analysis to use distributed lag models (DLMs) [32] or its generalization, distributed lag non-linear models (DLNMs) [21,31]. DLNM has been widely used to assess the association between meteorological variables and infectious diseases such as leishmaniasis [33], tuberculosis [34], pneumonia [35], and malaria [36,37,38,39,40]. Preliminary analysis based on Poisson generalized linear models indicated an overdispersion due to non-linear patterns and large variance; therefore, quasi-Poisson models were used. DLNM has an advantage of the flexibility to simultaneously explore the non-linear and delayed effects of meteorological factors on health outcomes [31,41,42].

The model is of the form:

(1) $$Y_{t}\sim quasiPoisson\left(\mu_{t}\right)$$

(2) $$log\left(\mu_{t}\right)=\alpha +\beta T_{ij,t-l}+\gamma R_{ij,t-l}+ns\left(RH_{ij,t-l},3\right)+ns\left(Time_{ij,t-l},7\right)$$

where $Y_{t}$ is the number of malaria cases on week *t* with mean $\mu_{t}$, α  is the model intercept; $T_{j}$, and $R_{j}$ represent the weekly average meteorological variables temperature and rainfall with their corresponding coefficients β and γ generated by the “cross-basis” function in the DLNM model defining the functional relationship between meteorological variables and the non-linear exposure-response curve; ns(.) is the natural cubic spline of relative humidity with 3 degrees of freedom (*df*). The model includes a cubic spline of relative humidity with 3 *df* and Time with 7 *df* per year to control for seasonal and long-term trends. The non-linear and delayed effect of temperature and rainfall was modelled through a bi-dimensional cross-basis function described by quadratic B-splines with 3 *df* for the exposure-response with three internal knots placed at equal distance and a linear function for the lag-response relationship. Previous malaria studies [36,37,38,39,40] have used a maximum lag of 10 to 15 weeks for meteorological variables. Hence, considering both the extrinsic and intrinsic incubation period of malaria parasites [30,43,44,45], which is key to the malaria vectors’ population dynamics, we used a maximum of 15 weeks for temperature and rainfall lags in this study.

The results of exposure-response associations were expressed in logarithms of relative risks (logRRs) [38,40], which were calculated with reference to the minimum incidence temperature (MIT) and rainfall (MIR). We defined MIT and MIR as the minimum temperature/rainfall with the lowest incidence of malaria. Further subgroup analyses investigated associations between weekly malaria counts for different age groups and gender.

Sensitivity analysis was performed to assess whether the model parameters were robust to changing *dfs* for exposure-response (3–5) and varying smooth functions. The results were robust to changing *dfs*; moreover, the model chosen was appropriate based on Akaike information criteria for quasi-Poisson (Q-AIC) (Supplementary Material Table S1).

All analyses were implemented with the package “dlnm” [46] in R statistics software v3.4.0 [47].

## 3. Results

### 3.1. Descriptive Analysis

A total of 2995 malaria cases were recorded between 6th February 2000 and 23rd November 2009 in Rajasthali, of which 58% were males, and most (93%) were less than 50 years of age. The average weekly mean, minimum, and maximum temperatures were 25.2 °C, 22.0 °C, and 31.1 °C, respectively. The mean weekly rainfall during the study period ranged from 0 mm to 686 mm with a mean of 47.8 mm. Weekly relative humidity ranged from 81% to 100%, with a mean of 95.5% (Table 1).

### 3.2. Association between Weather Variables and Malaria

**Figure 2:**
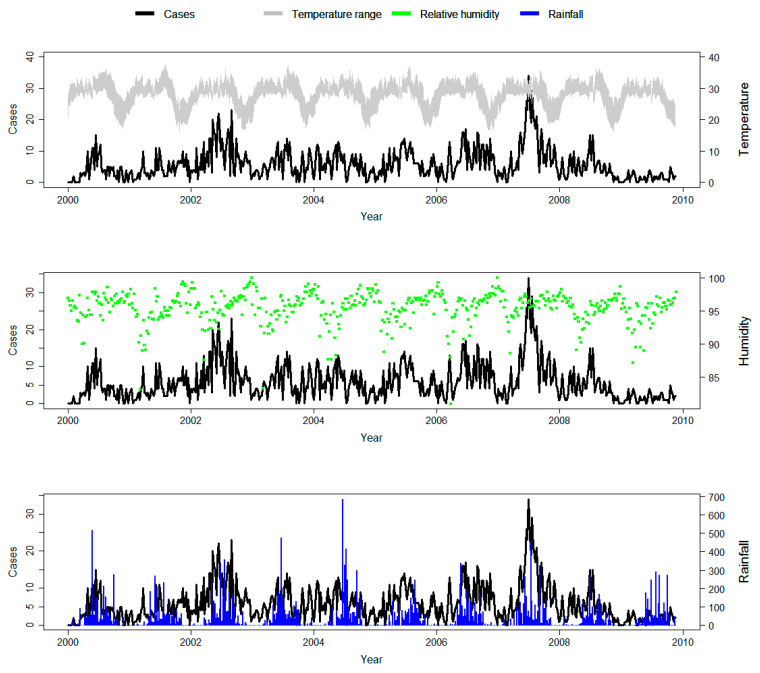
Time-series plots of weekly malaria cases, temperature range (weekly maximum to minimum), humidity, and rainfall in the study area.

Table 1 presents the Spearman correlation of climatic variables and malaria counts. Temperature (mean, minimum, and maximum temperatures) and rainfall were positively correlated with malaria (*p* < 0.001). Figure 2 presents the weekly time-series distribution of weather-malaria relationships. The figure revealed seasonal patterns of alternating highs and lows of malaria cases, which mirrored temperature and rainfall patterns, and inversely mirrored relative humidity (Figure 2 and Figure S1).

**Figure 3:**
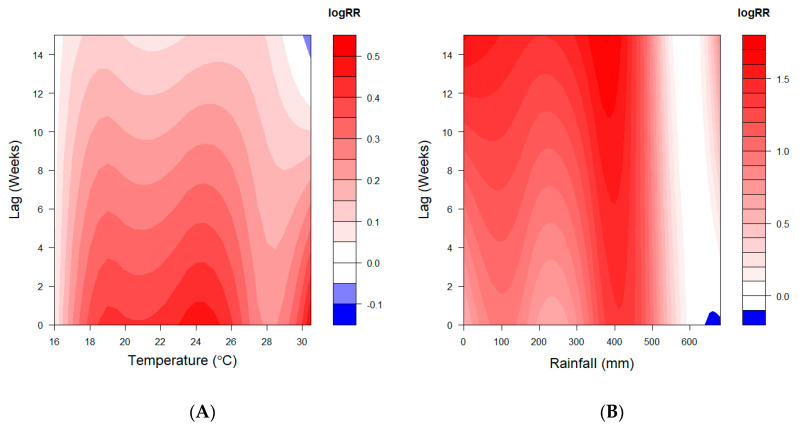
3-D plot for relative risks (RR) of malaria disease along, (**A**) temperature, (**B**) rainfall, and lags produced by DLNM for all ages. The logRR are estimated with reference to 15.8 °C mean temperature and 609 mm of rainfall.

As shown in the 3-D plot of the weather-lag-malaria relationship displayed in Figure 3, the associations were non-linear. The temperature-associated malaria risk was immediate (lag 0) at a higher temperature and diminished over longer lags. A temperature lower than the reference minimum incidence temperature, MIT = 15.6 °C (SD = 1.0 °C), reduces the risk of malaria infection. The effect of rainfall was higher at a rainfall value less than the reference minimum incidence rainfall of 609 mm (SD = 16.2 mm) and increased with lag weeks.

**Figure 4:**
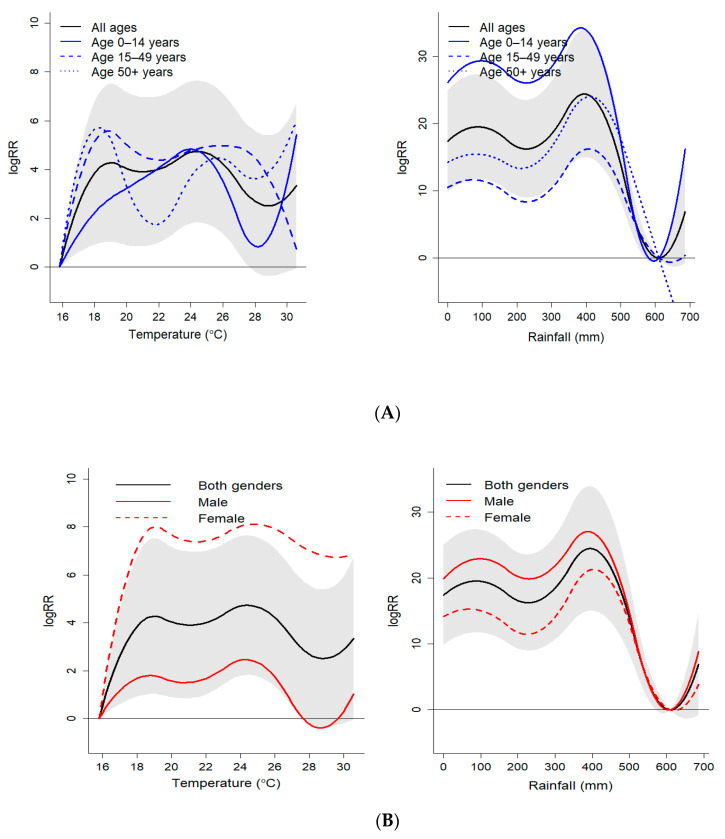
Overall association (relative risk) of temperature exposures with malaria over 0–15 lag weeks, by (**A**) age group and (**B**) gender in the study period. The grey shaded area represents the 95% CI for the estimated logRR for combined data (black line). The logRR are estimated with reference to 15.8 °C mean temperature and 609 mm of rainfall.

The cumulative effects (lag 0–15 weeks) of weather variables on malaria indicate that temperature and rainfall were associated with the incidence of malaria (Figure 4). Malaria risk associated with temperature was significantly higher for increasing temperatures >16 °C. Both temperature and rainfall curves revealed two peaks in the cumulative risk at 19 °C (logRR = 4.3, 95% CI: 1.1–7.5) and 24.5 °C (logRR = 4.7, 95% CI: 1.8–7.6) for temperature and at 86 mm (logRR = 19.5, 95% CI: 11.7–27.3) and 396 mm (logRR= 24.4, 95% CI: 15.0–33.9) for rainfall. The logRR at 90th and 97.5th percentile temperatures of 28.7 °C and 29.5 °C were 2.5 (95% CI: −0.4–5.4) and 2.6 (95% CI: −0.3–5.5), respectively (Table 2). For cumulative exposure (lag 0–15 weeks) to 90th percentile rainfall (130 mm), the logRR was 17.6 (95% CI: 9.9–25.1).

Lag-specific temperature- and rainfall-malaria associations for specific weather values are displayed in Figures S2 and S3, respectively. Increased and steep temperature-malaria associations were observed for higher temperature values and were highest at lag 0. The cumulative effects decreased with lag weeks. A positive lag associative effect was observed for rainfall, which increased with lag.

### 3.3. Sub-Group Analysis

The effects of temperature and rainfall on malaria incidence were investigated for different sub-populations. Table 2 and Figure 4 present the cumulative effects of various temperature and rainfall percentiles on the occurrence of malaria at lag 0–15 weeks for different sexes and age groups. We found that temperature significantly increased the risk of malaria for people 15–49 years. This association was consistent across the different percentiles of temperature. When we looked at the meteorological-malaria association between males and females, temperature had a stronger association with malaria incidence in females (logRR = 6.8, 95% CI: 3.1, 10.6) compared to males (logRR = −0.4 (95% CI: −3.6, 2.8)), especially at the 90th percentile (28.7 °C).

All sub-populations were susceptible to rainfall-associated malaria incidence. People aged <15 years and 50+ years were the most sensitive to rainfall with a very high risk of developing malaria (logRR = 29.0, 95% CI: 18.2, 39.8) and (logRR = 15.0, 95% CI: 4.34, 34.8), respectively, at cumulative exposure to the 90th percentile or 130 mm of rainfall. Men were more susceptible to malaria (logRR = 13.9, 95% CI: 5.5, 22.4) as compared to women (logRR = 10.4, 95% CI: 1.5, 19.3) at high rainfall (97th percentile or 274 mm).

## 4. Discussion

Our study has shown that the occurrence of malaria is significantly and positively associated with temperature and rainfall in the Chittagong Hill Tracts in Bangladesh. We found women to be more vulnerable to malaria at higher temperatures, while children and the elderly were more susceptible to malaria at increased rainfall.

The strong association observed in our study between environmental temperature and malaria occurrence could be explained by the high sensitivity of the *Anopheles* mosquitoes and *Plasmodium* parasites to temperature. Both the extrinsic and intrinsic incubation periods for malaria are characterized by high fluctuations and are dependent on the ambient temperature [48,49]. Increased temperature amplifies parasite replication and shortens the extrinsic incubation period while intensifying the mosquito biting rate. This results in a faster and increased rate of malaria parasite transmission from an infected person to a healthy individual. The incubation period (intrinsic) within the body of the newly infected individual can also vary and may be as short as four days [50]. Our data suggest that malaria incidence declined at a temperature below 15.6 °C. This finding is supported by studies demonstrating that the optimal breeding of mosquitoes and transmission of the *Plasmodium* malaria parasite occurs at temperatures between 23 °C and 33 °C [51,52]. The risks of malaria estimated at these temperatures were immediate (highest at lag 0). Kipruto and colleagues found that the risk of malaria associated with temperature increased with maximum temperatures at lag 0 and 1 months [53]. A study in South-West Ching showed that the risk of malaria associated with an increase in temperature was found to be highest at 4–6 lag weeks when daily fluctuations in temperature were low to moderate [54]. In another Southwestern China study, Wu et al. [40] reported that the risk of malaria increased after 8 weeks of temperatures above 28 °C, while Sewe et al. [39] reported that the effect of sunshine duration peaked at 5-week lag in Guangdong, China.

The positive association that we found between rainfall and malaria occurrence could be attributed to more favourable breeding sites resulting from rain [9,55,56]. We also found that the association of rainfall with malaria was delayed for a few weeks (increase with lag) up to a peak of 396 mm cumulative rainfall. A previous study [40] suggested that significant interaction exists between lagged rainfalls and malaria in the South-West of China. The authors found low (<1.6 mm) and medium (1.6–20.0 mm) weekly rainfall levels at the 4th week to be associated with increased malaria risk along with increasing rainfall at other lags (up to 12 weeks); however, excessive rainfall decreases this risk [40]. Similarly, malaria risks have been found to increase with rain at longer lags, especially at moderate rain. For example, malaria risk was highest at a 2-month lag of precipitation in lowland and riverine areas of Baringo County, Kenya [53], and at a 12-week lag of 80 mm rainfall in three Western Kenyan districts [39]. Similarly, risks at a 5-week lag of 10 mm rainfall in Republic of Korea have been reported [38]. In Guangdong, China, the risk of malaria peaked at an 8-week lag of 150 mm/week rainfall [37]. A similar result was obtained in South-West China at much lower rainfall (0.1–13.1 mm), peaking at 11–12th weeks [36]. It is common knowledge that inadequate water management or delayed emptying of water pools provides breeding sites for mosquitoes over time and increases the probability of biting and malaria infection. Rainfall could also restrict the movement of individuals and result in more exposure to malaria vectors and parasites. Our study has shown that malaria occurrence starts to decline when rainfall exceeds 686 mm, which may be due to the heavy rain flushing out the mosquito breeding sites [57]. However, another study conducted around the same time period and in the same Chittagong Hill Tracts area did not find a significant association between temperature, rainfall, humidity, and malaria [4]. One key difference between that study and ours is that the investigators of the other study used a lag of 0–3 months, whereas, in our research, we used weekly lags. 

A key finding of our study is that there is a stronger and positive association between ambient temperature and malaria incidence in women. This finding could have significant public health implications. However, before we go into any policy recommendations, the plausibility of this finding needs to be explained and established by taking both biological susceptibility and vulnerability of women in the local context into account. Pregnant women are found to be more susceptible to malaria because of their reduced immune response to infections [58,59,60]. However, to our knowledge, there is no available evidence supporting increased susceptibility of women to malaria in increased temperature. A study from China found females at a higher risk of sunshine and precipitation-related malaria, but no gender difference in the sensitivity to temperature-associated malaria risk was observed [37]. The increased risk of malaria associated with temperature observed among women in this study could be related to the socio-cultural norms and occupations of women specific to our study communities, which are mostly tribal and, unlike the rest of the country, have unique matriarchal societies [61]. Women in these indigenous communities are involved in Jhum (slash and burn) cultivation, which has been found to be a risk factor for malaria infection [61]. However, this alone could not make women more vulnerable to the disease as both men and women equally participate in Jhum cultivation. Moreover, in increased temperature, men working outdoors are more likely to expose their body to cope with the heat, which makes them more vulnerable to mosquito bites [62]. In regard to indoor and household activities, women are more at risk of mosquito bites by waking up early in the morning to perform household chores [63]. This risk of exposure could be increased on hot summer days when women start doing their household chores even earlier.

Similarly, care-seeking behaviour could also explain the increased malaria-climate risk for women. In low-resource settings, women are found to be more likely than similar-aged men to walk long distances to obtain malaria treatment from a clinic [64]. Similar kinds of care-seeking behaviour could also have happened in our study area. This determination and these differences in care-seeking by gender might even be stronger during the hot summer months. Moreover, in the matriarchal society of our study area, women would enjoy more freedom in decision-making regarding care-seeking for their febrile illnesses. Our analysis, based on quantitative data, could not establish these socio-cultural circumstances as factors putting women more at risk of malaria infection with increased temperature. We also could not determine whether there was variation in the use of bed nets by women as compared to men in the family, which could further be modified due to the rise of the ambient temperature. This could put women more at risk of mosquito bites in warmer weather. Further investigations, involving in-depth explorations using qualitative methods, are thus needed to establish this finding. It is important to go back to those tribal communities and obtain their insiders’ view.

Although our study area is unique because of its geographical and socio-cultural structure, and the findings from our study may not be generalizable to the whole of Bangladesh, it is important to understand this finding, as the Chittagong Hill Tract districts are responsible for the major burden of malaria in the country. Additionally, our findings can be generalizable to similar geographic and socio-cultural settings in other parts of the world.

The influence of rainfall on malaria incidence was more remarkable for children and the elderly, which has significant public health implications. This finding is consistent with those obtained in adults in Cambodia [65] and African children [66]. Previous studies have linked malaria incidence to occupation, such as agriculture and forestry in Bangladesh, Afghanistan, Cambodia, and Indonesia [2,3,9,66]. A large proportion (40–50%) of Chittagong Hill Tract’s population engages in agricultural activities [67], which could expose these populations to the vectors [33,61]. However, occupation could not explain our findings of a higher vulnerability of children and the elderly to malaria with rainfall. Our result is consistent with a Ugandan study assessing malaria prevalence in relation to rainfall that showed higher mean parasite density observed in children with increased rain [37,68]. The authors suggested that a less developed immune system and incapability to clear parasites more effectively as the reasons behind these findings. A study from China also demonstrated that young children and the elderly were more susceptible to temperature-, sunshine-, and precipitation-associated malaria [37]. For children, the authors also referred to their vulnerable immune system, and to a higher likelihood of experiencing mosquito bites as the most likely factors contributing to children’s increased susceptibility. Loss of physical function and poorer general health were thought to be the factors behind putting the elderly at higher risk of weather-related malaria. Our observed increased risk of malaria associated with rainfall in children and the elderly could also partly be due to the restriction of movements among children and the elderly during heavy rain, putting them more at risk of mosquito bites. However, that needs to be further investigated. 

This study has a few limitations. First, we considered only the association between climatic variables and malaria, but there are potentially other risk factors for malaria. Second, we did not clearly distinguish between malaria parasites in the aggregated malaria data. However, this would not significantly affect the findings in this study as data from a previous nationwide malaria prevalence survey showed around 90% of the parasites were *Plasmodium falciparum,* about *5% Plasmodium vivax*, and the remaining 5% were mixed infections with these two species [11]. Our study findings are thus more generalizable to a population mostly endemic with *P. falciparum,* which is also the most deadly and drug-resistant. Moreover, asymptomatic carriage of *P. falciparum* over time with some variations could also influence the transmission of the disease. Third, there are often challenges in validating the exposure-lag-response smooth functions employed in DLNM [69].

The strengths of our study include the use of a robust set of data directly extracted from hospital records by our study staff visiting the sub-district health complexes in Rajasthali. This allowed us to obtain individual-level data with age and sex distribution and the dates of each hospital visit. Our data set is thus more informative as compared to the monthly aggregated numbers reported through the passive surveillance system in place to report malaria incidence in Bangladesh. Additionally, we microscopically confirmed all malaria diagnosis to reduce any chance of misclassification in our data. Finally, the weather station for that region is based in Rangamati district. Hence, the weather data used in our analyses thus represent climatic variability of our study area directly.

It is worth mentioning that the bimodal relationship between temperature/rainfall and malaria observed in this study deserved further investigation. Previous studies in Kenya [70] and Colombia [71] demonstrate a unimodal nature of the risk curve. In this study, the bimodal relationship is more prominent in elderly, which may also influence the overall risk.

## 5. Conclusions

Our study presents a crucial addition to prior knowledge of malaria-climate association in the Chittagong Hill Tracts of Bangladesh and for similar environment and topography in the region. The significant positive association between temperature and rainfall, and malaria, further established in this study foresees changes in the distribution of malaria in the country and the region due to global warming. This warrants that the health systems be better prepared for an increasing burden and varying spread of malaria. The association observed between climate variabilities and the incidence of malaria will help to better time the control measures and prepare the health system for managing any upsurge of malaria cases at a local level. Identifying women, children, and the elderly as more vulnerable and susceptible groups to malaria as climate changes is significant, albeit a partially unexplained finding from this study. This needs to be further investigated while updating current programmes and policies to address the needs of these vulnerable groups. Our study has significant implications for malaria control programmes in Bangladesh and the region. 

## Figures and Tables

**Table 1:** Descriptive summaries of weekly malaria cases and meteorological factors in Rajasthali, 2000–2009.

Variables	Mean	SD	Min	Percentile			Max	Cor. (*p*-Value) *
				25	50	75		
Malaria Cases	5.7	4.9	0	2	4	8	34	
Mean Temperature (°C)	25.2	3.7	15.8	22.0	26.8	28.0	30.7	0.32 (<0.001)
Minimum Temperature (°C)	21.9	4.2	11.2	18.2	24.1	25.3	27.8	0.34 (<0.001)
Maximum Temperature (°C)	31.1	2.8	23.1	29.4	31.7	33.1	37.8	0.21 (<0.001)
Rainfall (mm)	47.8	79.5	0.0	0.0	16.0	61.0	686.0	0.16 (<0.001)
Relative Humidity (%)	95.5	2.4	81.0	94.4	96.0	97.0	100.0	−0.02 (0.704)

* Correlation (Spearman correlation coefficient) between cases and meteorological factors.

**Table 2:** Overall cumulative association (logarithms of relative risks (logRR) ^†^, 95% CI) for malaria-temperature/rainfall distributed lag non-linear models (DLNM) for temperature percentiles and by sex and age.

Characteristics	Overall N = 2995	Age Group, *n* (%)			Gender, *n* (%)	
		0–14 Years	15–49 Years	50 + Years	Male	Female
		1381(45.1)	1424(47.6)	190(6.3)	1740(58.1)	1255(41.9)
Temperature						
2.5th (17.3 °C)	3.1(0.7, 5.5)	1.7(−1.5, 4.8)	4.2(1.4, 6.9)	4.9(−1.1, 10.6)	1.4(−1.3, 4.0)	5.6(2.6, 8.7)
10th (18.9 °C)	4.2(1.1, 7.5)	2.8(−1.5, 7.2)	5.6(1.8, 9.4)	5.3(−2.8, 13.6)	1.8(−1.9, 5.4)	7.9(3.7, 12.8)
90th (28.7 °C)	2.5(−0.4, 5.4)	1.0(−2.8, 4.9)	3.9(0.5, 7.2)	4.4(−3.6, 11.2)	−0.4(−3.6, 2.8)	6.8(3.1, 10.6)
97.5th (29.5 °C)	2.6(−0.3, 5.6)	2.2(−1.7, 6.1)	2.7(−0.7, 6.2)	4.8(−2.5, 12.0)	−0.1(−3.4, 3.5)	6.7(2.9, 10.5)
Rainfall						
10th (0 mm)	17.3(9.8, 25.0)	26.2(15.5, 36.8)	10.5(1.8, 19.1)	14.2(−4.8, 33.2)	19.9(11.4, 28.3)	14.1(4.4, 23.1)
90th (130 mm)	19.1(11.4, 26.7)	29.0(18.3, 39.8)	10.6(2.1, 19.8)	15.0(4.3, 34.3)	22.6(13.9, 31.2)	14.4(4.5, 24.2)
97.5th (274 mm)	17.2(9.6, 24.3)	17.2(16.5, 37.8)	9.2(0.6, 17.8)	14.9(−3.9, 33.7)	20.7(12.3, 29.1)	12.6(2.9, 22.3)

^†^ logRR estimates at temperature/rainfall percentiles: 2.5th percentile (°C); 10th percentile (°C); 90th percentile (°C); 97.5th percentile (°C) for the overall lag exposures 0–15 weeks.

## Supplementary Materials

The following are available online at https://www.mdpi.com/1660-4601/17/24/9469/s1, Figure S1. Decomposition of the malaria data, Figure S2. Lag (in weeks) specific associations (outcomes = logRR) for various temperature values (var), Figure S3. Lag specific associations for various rainfall values (var), Table S1. Selection of the best model, exploring various exposure-response and lag-response functions and degrees of freedom 3–5 at maximum lag 15.
