# Supplementary material for: Disparities in Risks of Malaria Associated with Climatic Variability among Women, Children and Elderly in the Chittagong Hill Tracts of Bangladesh
Source: Int J Environ Res Public Health. 2020 Dec 17;17(24):9469. doi: 10.3390/ijerph17249469 (PMC7766360; doi:10.3390/ijerph17249469)
Supplement: Supplementary file 1 [file ijerph-17-09469-s001.pdf]

Supplementary Materials:

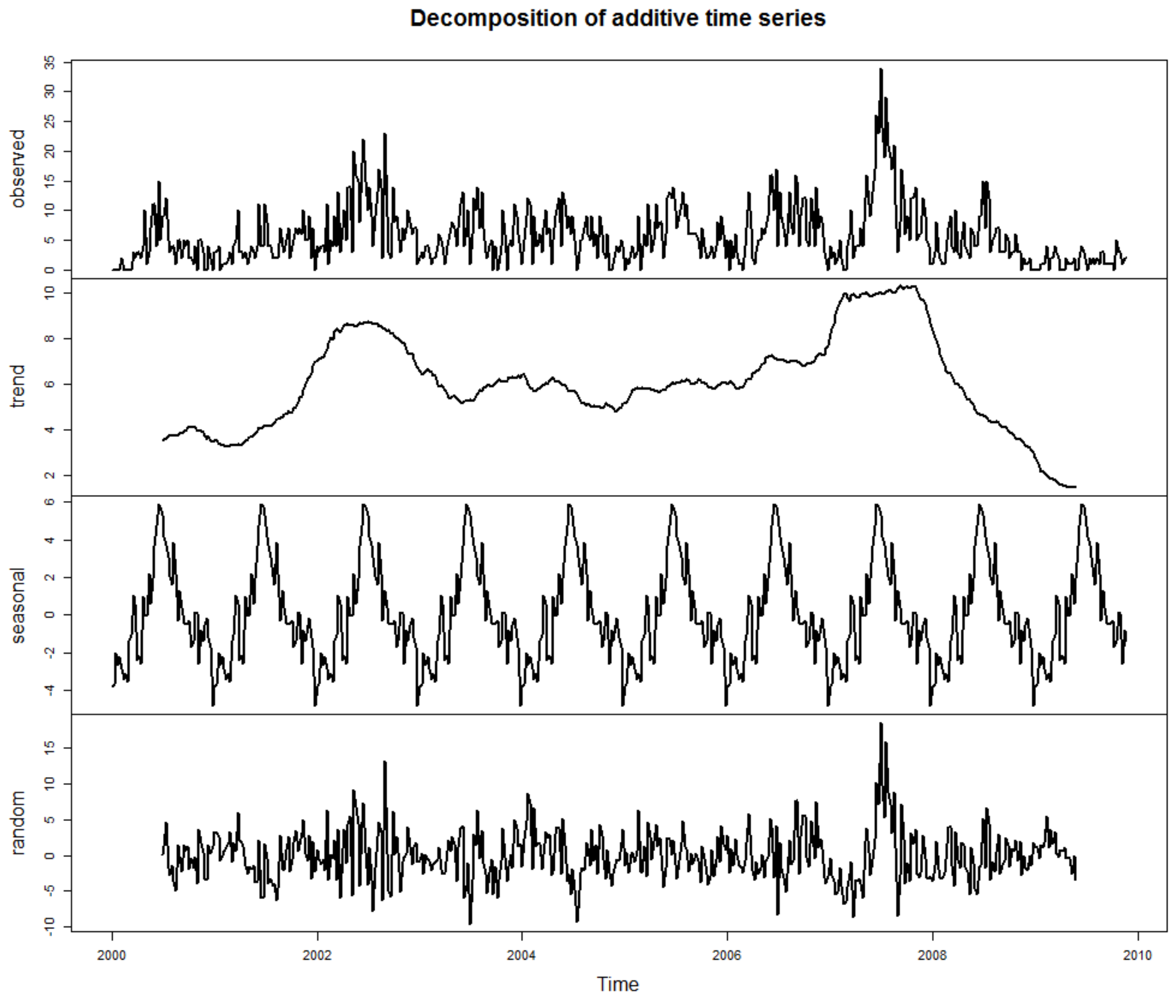

Figure S1. Decomposition of the malaria data.

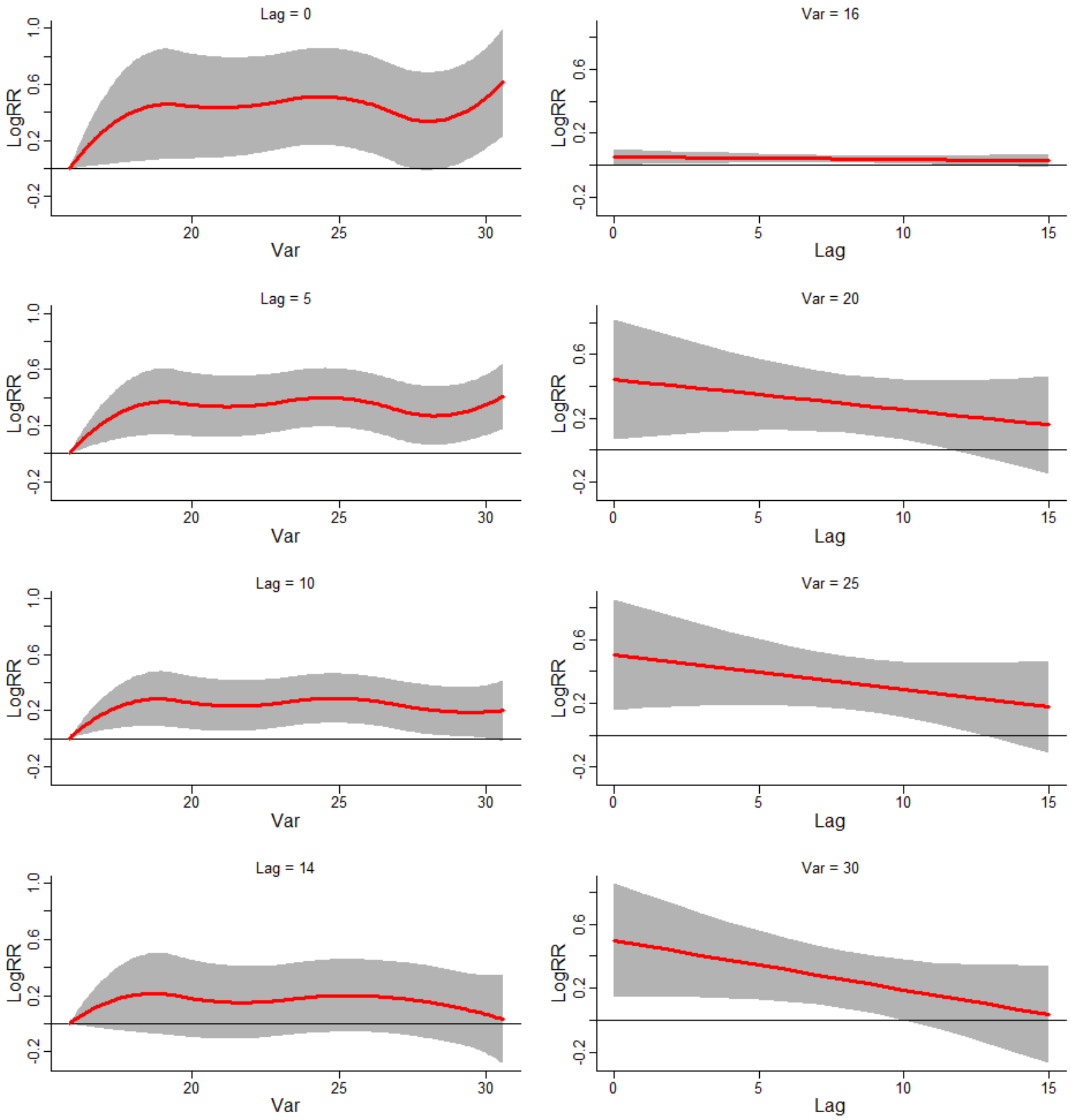

Figure S2. Lag (in weeks) specific associations (outcomes=logRR) for various temperature values (var).

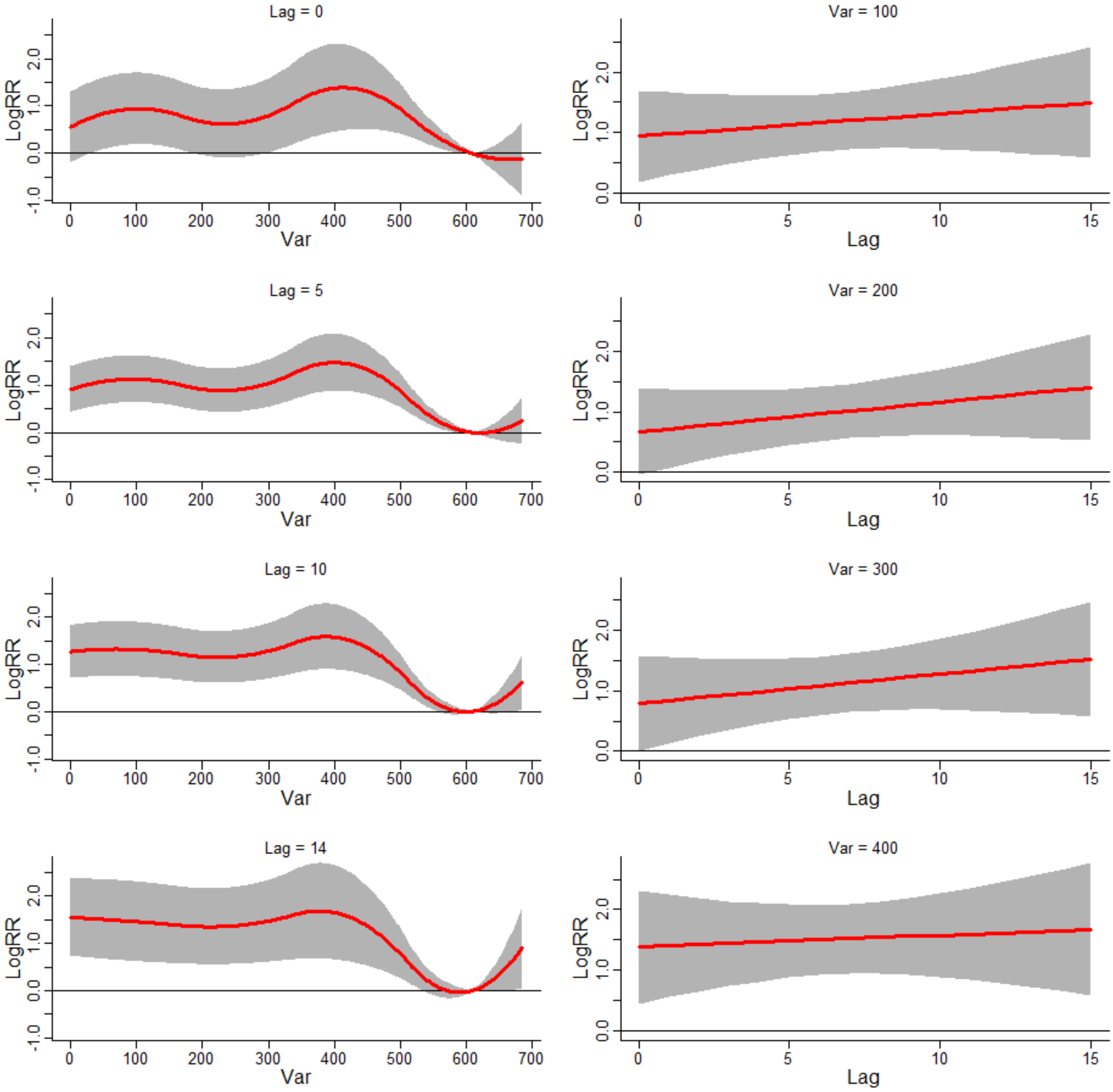

Figure S3. Lag specific associations for various rainfall values (var).

Table S1. Selection of the best model, exploring various exposure-response and lag-response functions and degrees of freedom 3-5 at maximum lag 15.

	Temperature -Response	Rainfall -Response	Lag-Response	Total df	QAIC
Model 1	Linear	Linear	strata	1	3317.5
Model 2	Quadratic B-spline	Linear	strata	3	3279.5
Model 3	Quadratic B-spline	Linear	strata	4	3286
Model 4	Quadratic B-spline	Linear	strata	5	3284.1
Model 5	Linear	Linear	Linear	2	3317.7
Model 6	Quadratic B-spline	Linear	Linear	6	3258.9
Model 7	Quadratic B-spline	Linear	Linear	8	3266.1
Model 8	Quadratic B-spline	Linear	Linear	10	3271.1
Model 9	Linear	Linear	Natural spline	5	3325
Model 10	Quadratic B-spline	Linear	Natural spline	15	3267.1
Model 11	Quadratic B-spline	Linear	Natural spline	20	3294.4
Model 12	Quadratic B-spline	Linear	Natural spline	25	3306.7
Model 13	Linear	Linear	Quadratic B-spline	5	3328.4
Model 14	Quadratic B-spline	Linear	Quadratic B-spline	15	3269.4
Model 15	Quadratic B-spline	Linear	Quadratic B-spline	20	3297.2
Model 16	Quadratic B-spline	Linear	Quadratic B-spline	25	3310.6
Model 17	Linear	Quadratic B-spline	strata	1	3291.8
Model 18	Quadratic B-spline	Quadratic B-spline	strata	3	3267.6
Model 19	Quadratic B-spline	Quadratic B-spline	strata	4	3269.1
Model 20	Quadratic B-spline	Quadratic B-spline	strata	5	3256.4
Model 21	Linear	Quadratic B-spline	Linear	2	3287.6
Model 22	Quadratic B-spline	Quadratic B-spline	Linear	6	3238.6
Model 23	Quadratic B-spline	Quadratic B-spline	Linear	8	3235.4
Model 24	Quadratic B-spline	Quadratic B-spline	Linear	10	3233.7
Model 25	Linear	Quadratic B-spline	Natural spline	5	3265.4
Model 26	Quadratic B-spline	Quadratic B-spline	Natural spline	15	3225.2
Model 27	Quadratic B-spline	Quadratic B-spline	Natural spline	20	3240.4
Model 28	Quadratic B-spline	Quadratic B-spline	Natural spline	25	3250.1
Model 29	Linear	Quadratic B-spline	Quadratic B-spline	5	3264.8
Model 30	Quadratic B-spline	Quadratic B-spline	Quadratic B-spline	15	3222.2
Model 31	Quadratic B-spline	Quadratic B-spline	Quadratic B-spline	20	3239.3
Model 32	Quadratic B-spline	Quadratic B-spline	Quadratic B-spline	25	3251.4
Model 33	Linear	Quadratic B-spline	strata	1	3297.9
Model 34	Quadratic B-spline	Quadratic B-spline	strata	3	3276.2
Model 35	Quadratic B-spline	Quadratic B-spline	strata	4	3276.9
Model 36	Quadratic B-spline	Quadratic B-spline	strata	5	3262.1
Model 37	Linear	Quadratic B-spline	Linear	2	3263.9
Model 38	Quadratic B-spline	Quadratic B-spline	Linear	6	3218.4
Model 39	Quadratic B-spline	Quadratic B-spline	Linear	8	3210.4

Model 40	Quadratic B-spline	Quadratic B-spline	Linear	10	3199.5
Model 41	Linear	Quadratic B-spline	Natural spline	5	3254.2
Model 42	Quadratic B-spline	Quadratic B-spline	Natural spline	15	3216
Model 43	Quadratic B-spline	Quadratic B-spline	Natural spline	20	3227.8
Model 44	Quadratic B-spline	Quadratic B-spline	Natural spline	25	3228.6
Model 45	Linear	Quadratic B-spline	Quadratic B-spline	5	3261
Model 46	Quadratic B-spline	Quadratic B-spline	Quadratic B-spline	15	3221.1
Model 47	Quadratic B-spline	Quadratic B-spline	Quadratic B-spline	20	3234
Model 48	Quadratic B-spline	Quadratic B-spline	Quadratic B-spline	25	3242.1
Model 49	Linear	Quadratic B-spline	strata	1	3187.6
Model 50	Quadratic B-spline	Quadratic B-spline	strata	3	3177.5
Model 51	Quadratic B-spline	Quadratic B-spline	strata	4	3176.5
Model 52	Quadratic B-spline	Quadratic B-spline	strata	5	3155.6
Model 53	Linear	Quadratic B-spline	Linear	2	3167.3
Model 54	Quadratic B-spline	Quadratic B-spline	Linear	6	3135.8
Model 55	Quadratic B-spline	Quadratic B-spline	Linear	8	3129.3
**Model 56**	**Quadratic B-spline**	**Quadratic B-spline**	**Linear**	**10**	**3110.7**
Model 57	Linear	Quadratic B-spline	Natural spline	5	3166.6
Model 58	Quadratic B-spline	Quadratic B-spline	Natural spline	15	3138.5
Model 59	Quadratic B-spline	Quadratic B-spline	Natural spline	20	3151
Model 60	Quadratic B-spline	Quadratic B-spline	Natural spline	25	3145.2
Model 61	Linear	Quadratic B-spline	Quadratic B-spline	5	3190.6
Model 62	Quadratic B-spline	Quadratic B-spline	Quadratic B-spline	15	3160.7
Model 63	Quadratic B-spline	Quadratic B-spline	Quadratic B-spline	20	3175.2
Model 64	Quadratic B-spline	Quadratic B-spline	Quadratic B-spline	25	3173.5
